# Tissue factor links inflammation, thrombosis, and senescence in COVID-19

**DOI:** 10.1038/s41598-022-23950-y

**Published:** 2022-11-18

**Authors:** Dayna Nguyen, Hye-Min Jeon, Jeongwu Lee

**Affiliations:** Department of Cancer Biology, Lerner Research Institute, Cleveland Clinic, Cleveland, OH USA

**Keywords:** Infectious diseases, Computational biology and bioinformatics, Immunology, Diseases, Medical research

## Abstract

COVID-19 is a highly contagious respiratory infection caused by the SARS-CoV-2 virus. The infected lung epithelial cells secrete a group of chemokines and cytokines, which triggers harmful cytokine storms and hyper-thrombotic responses. Recent studies have proposed that viral-induced senescence is responsible for cytokine release and inflammation in COVID-19 patients. However, it is unknown whether cellular senescence is commonly triggered after viral infection and how inflammation and thrombosis, hyper-activated in these patients, are functionally connected. To address these questions, we conducted a bioinformatics-based meta-analysis using single-cell and bulk RNA sequencing datasets obtained from human patient studies, animal models, and cell lines infected with SARS-CoV-2 and other respiratory viruses. We found that the senescence phenotype is robustly upregulated in most SARS-CoV-2-infected patients, especially in the infected lung epithelial cells. Notably, the upregulation of Tissue factor (F3), a key initiator of the extrinsic blood coagulation pathway, occurs concurrently with the upregulation of the senescence-associated secretory phenotype (SASP) factors. Furthermore, F3 levels are positively correlated with the senescence and hyper-coagulation gene signatures in COVID-19 patients. Together, these data demonstrate the prevalence of senescence in respiratory viral infection and suggest F3 as a critical link between inflammation, thrombosis, and senescence in these disease states.

## Introduction

The coronavirus 19 (COVID-19) pandemic has led to devastating impacts on global health and economies. The viral strain behind COVID-19, severe acute respiratory syndrome coronavirus 2 (SARS-CoV-2), infects the upper respiratory airways and can induce respiratory syndromes ranging from mild upper airway resistance to lethal pneumonia^1,2^. SARS-CoV-2 infects type II epithelial alveolar cells by binding its S protein to angiotensin-converting enzyme 2 (ACE2) receptors^3^. Upon infection, the patient undergoes several biological responses including an inflammatory immune response and a thrombotic response.

SARS-CoV-2 can disrupt normal immune responses^4^. In severe patients with COVID-19, the immune system is impaired by lymphopenia and monocyte/granulocyte abnormalities^4,5^. Simultaneously, severe cases have an excessive inflammatory response marked by sharp increases in cytokine and antibody production^4^. The sharp increase in cytokines can cause the cytokine storm, which leads to further inflammation, tissue damage, and ultimately multi-organ failure^6^. Thus, the excessive inflammatory response is a leading factor in COVID-19 mortality^7^.

The thrombotic response is a concurrent reaction to SARS-CoV-2. Severe COVID-19 patients demonstrate increased thrombosis and coagulation activities^8,9^. Autopsy results have noted frequent vascular fibrinous thrombi in the lungs and other organs of critically ill COVID-19 patients^8,9^. In hemostatic tests, severe COVID-19 cases show increased D-dimer, prothrombin time (PT), activated partial thromboplastin time (aPTT), fibrinogen, and factor VIII^9^. These factors contribute to the formation of pulmonary embolism and deep vein thrombosis, two of the most frequently noted thrombotic events in severe COVID-19 patients^8,9^. Furthermore, the thrombotic response can exacerbate the severity of COVID in those with comorbidities such as heart failure, hypertension, and chronic pulmonary obstructive disorder (COPD)^10^.

Recent studies by Lee et al*.* (2021) and Camell et al*.* (2021) have proposed that senescence exacerbates the cytokine storm and inflammatory response in COVID-19 patients^11,12^. Senescence is induced by various external and internal stressors including activation of oncogene, DNA damage, tissue damage, aging, and viral infection^11,13^. Once initiated, senescence elicits wide-ranging changes including gene expression, epigenetic remodeling, and alterations in metabolic states^11–13^. Cellular senescence is defined as a state of cell cycle arrest and secretion of multiple cytokines and chemokines, referred to as the senescence-associated secretory phenotype (SASP)^13^. These SASP factors include many pro-inflammatory factors, chemokines, and pro-thrombotic proteins^14^. Dysregulation and over-activation of these inflammatory SASP factors lead to deleterious physiological effects and are proposed to contribute to the severe inflammatory responses of COVID-19^8,15^. For example, Interleukin-6 (IL6) and tumor necrotic factor alpha (TNFα), two representative SASP factors, are the main contributors to the hyperactive cytokine storm^6^. Interferon gamma (IFNγ), another SASP factor, is crucial for priming macrophage activation^16^. Thus, acute increases in the senescent cell population and/or SASP factors can be responsible for the mortality associated with severe COVID-19 cases^8,15^.

Although a few recent publications have reported that viral-induced senescence (VIS) is a critical mechanism leading to aggressive inflammation in severe COVID-19 patients^11,12^, it remains unclear whether senescence is a common event in COVID-19 patients. It is also incompletely understood why thrombosis is concurrently deregulated in these patients. To address this knowledge gap, we investigated the potential roles of senescence and F3 in viral infection models including the independent COVID-19 patient datasets. By analyzing the single-cell and bulk-RNA sequencing data, we aim to assess the prevalence of VIS and determine whether F3 signaling instigates the inflammatory, thrombotic, and senescent responses in COVID-19.

## Results

### Senescence is a common biological response to COVID-19 infection

VIS has been proposed to trigger the hyperactive inflammatory response in a subset of severe COVID-19 patients^12^. To confirm the above hypothesis in a broad range of COVID-19 cases, we analyzed the single-cell profiles obtained from multiple independent datasets. First, we analyzed the single-cell RNA-seq data of the bronchoalveolar lavage fluid (BALF) cells derived from 12 individuals; 3 mild and 6 severe COVID-positive, and 3 healthy control individuals (GSE145926)^17^. Three out of 9 COVID-positive patients had chronic diseases and two patients died in the hospital. All infected patients were treated with antiviral and anti-inflammatory interventions^17^.

**Figure 1:**
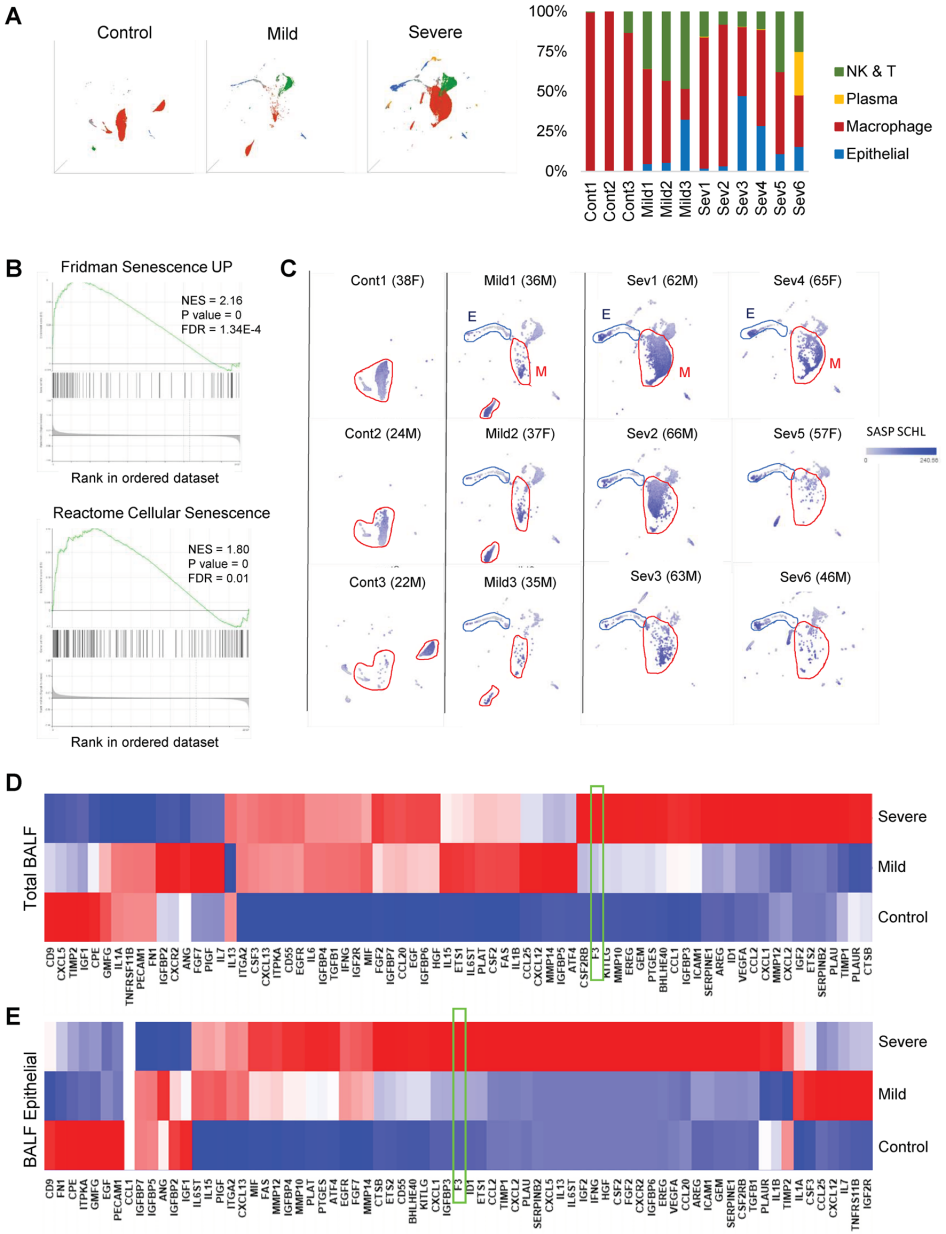
Senescence is a common response to COVID-19 infection. (**A**) UMAP projection of the major cell types in bronchoalveolar lavage fluid (BALF) cell population from healthy controls and COVID-19 patients (Control, *n* = 3; Mild, *n* = 3; Severe, *n* = 6). Each point represents a single cell. Major cell types were marked with different colors and quantitated per individual samples. (**B**) Representative GSEA plots of Reactome Cellular Senescence and Fridman Senescence UP gene sets. Positive normalized enrichment score (NES) indicates enrichment compared to the controls. (**C**) UMAP representation of BALF cell population from each COVID-19 patient. Each gray point represents a single cell. Age and sex were listed next to sample name. SASP factor expression levels using the SASP Schleigh gene list^39^ are marked with a blue color gradient. Prominent cell types are outlined and labeled. E = Epithelial cells and M = Macrophage. (**D**) Heat map plots of the SASP gene sets in BALF cells populations (Severe COVID patients: 41,323, Mild COVID patients: 8613, healthy control: 23,486 cells). (**E**) Heat map plots of the SASP gene sets in BALF epithelial cells (Severe: 2705, Mild: 551, healthy control: 72 cells). Note that F3 is not on the SASP SCHL list but added for comparison (boxed in green).

To determine what cell types were present in the patient samples, we used representative cell type-specific markers to profile the BALF cell populations (Fig. 1a). Most BALF cells from healthy control individuals were macrophages (92%). In contrast, COVID-19-infected patients had more diverse cell types within their BALF cell populations, with a notable increase in epithelial cells (about 6%) and Natural killer (NK)/T cells (about 17%) while macrophages are about 60 to 70%. First, we surveyed the status of cellular pathways that were significantly enriched in the BALF cells from COVID-positive patients in comparison to those from healthy controls by performing Gene Set Enrichment Analysis (GSEA)^18,19^. The top upregulated pathways in the BALF from COVID-19-infected patients include SARS-CoV-2 infection, cytokine response (GOBP Response to cytokine, KEGG Cytokine-cytokine receptor interaction), thrombosis (HP arterial thrombosis, GOBP Positive regulation of coagulation, GOBP coagulation), and senescence (Fridman senescence UP, Reactome cellular senescence) (Fig. 1b, Table 1). In addition, chemokine signaling and NF-kappa B signaling pathways, two pathways directly linked to senescence, are among the highly enriched pathways in COVID patient-derived BALF cell populations^14^ (Table 1).

**Table 1:** KEGG pathway analysis of BALF cells derived from COVID-19 patients compared to those from healthy controls. Ranked in order of normalized enrichment score (NES).

	Gene set ID	Gene set description	Gene set size	Enrichment score	Normalized enrichment score	*P* value	FDR
1	path:hsa04062	Chemokine signaling pathway	178	6.38E − 01	2.86E + 00	0.00E + 00	0.00E + 00
2	path:hsa04064	NF-kappa B signaling pathway	98	7.20E − 01	2.85E + 00	0.00E + 00	0.00E + 00
3	path:hsa04061	Viral protein interaction with cytokine and cytokine receptor	93	7.27E − 01	2.84E + 00	0.00E + 00	0.00E + 00
4	path:hsa04060	Cytokine-cytokine receptor interaction	253	5.95E − 01	2.79E + 00	0.00E + 00	0.00E + 00
5	path:hsa04620	Toll-like receptor signaling pathway	100	7.23E − 01	2.65E + 00	0.00E + 00	0.00E + 00
6	path:hsa04630	JAK-STAT signaling pathway	148	6.03E − 01	2.64E + 00	0.00E + 00	0.00E + 00
7	path:hsa04657	IL-17 signaling pathway	85	6.60E − 01	2.60E + 00	0.00E + 00	0.00E + 00
8	path:hsa05162	Measles	135	6.92E − 01	2.58E + 00	0.00E + 00	0.00E + 00
9	path:hsa05163	Human cytomegalovirus infection	216	6.25E − 01	2.57E + 00	0.00E + 00	0.00E + 00
10	path:hsa04622	RIG-I-like receptor signaling pathway	67	7.12E − 01	2.55E + 00	0.00E + 00	0.00E + 00
11	path:hsa05160	Hepatitis C	148	6.74E − 01	2.54E + 00	0.00E + 00	0.00E + 00
12	path:hsa04668	TNF signaling pathway	109	6.52E − 01	2.53E + 00	0.00E + 00	0.00E + 00
13	path:hsa04621	NOD-like receptor signaling pathway	164	6.67E − 01	2.52E + 00	0.00E + 00	0.00E + 00
14	path:hsa05161	Hepatitis B	159	6.44E − 01	2.49E + 00	0.00E + 00	0.00E + 00
15	path:hsa05203	Viral carcinogenesis	191	6.22E − 01	2.49E + 00	0.00E + 00	0.00E + 00
57	path:hsa04218	Cellular senescence	154	5.16E − 01	2.03E + 00	0.00E + 00	7.47E − 04

To investigate which cell types were responsible for the upregulation of the senescence pathway, we created UMAP single-cell profiles for each COVID-19-infected patient and clustered the single cells based on their cell types. Then, we inferred the level of SASP factor expression in each cell by color gradient (Fig. 1c). Moderate levels of SASP factor expression were noted in macrophage populations from both control and COVID patients. Notably, BALF epithelial cells of severe patients show a particularly strong SASP upregulation in comparison to healthy controls. These findings are consistent with the fact that SARS-CoV-2 virus infects lung epithelial cells via ACE2 receptor^3^.

We then aimed to quantitate which SASP factors were upregulated in the BALF cell population from COVID patients. Only a subset of genes can be reliably quantitated through single-cell RNA sequencing data. To account for this gene dropout, we converted single-cell profiles to pseudo-bulk profiles in which gene expression profiles of the clustered single-cell subpopulations are pooled. Using this method, we created three pseudo-bulk RNA profiles of total BALF cells derived from healthy control, mild COVID patients, and severe COVID patients. Heatmap plot analysis indicates that about 80% of representative SASP factor genes were upregulated in response to COVID-19 infection, with further upregulation in severe COVID patients (Fig. 1d). Next, we focused on the epithelial cells from the total BALF population (Fig. 1e). Our heatmap analysis showed that an even larger proportion of SASP signature genes were strongly upregulated in BALF epithelial cells from severe COVID patients (Fig. 1e). These findings demonstrate a strong positive correlation between the senescence phenotype and COVID-19 infection.

### F3 expression in COVID-19

**Figure 2:**
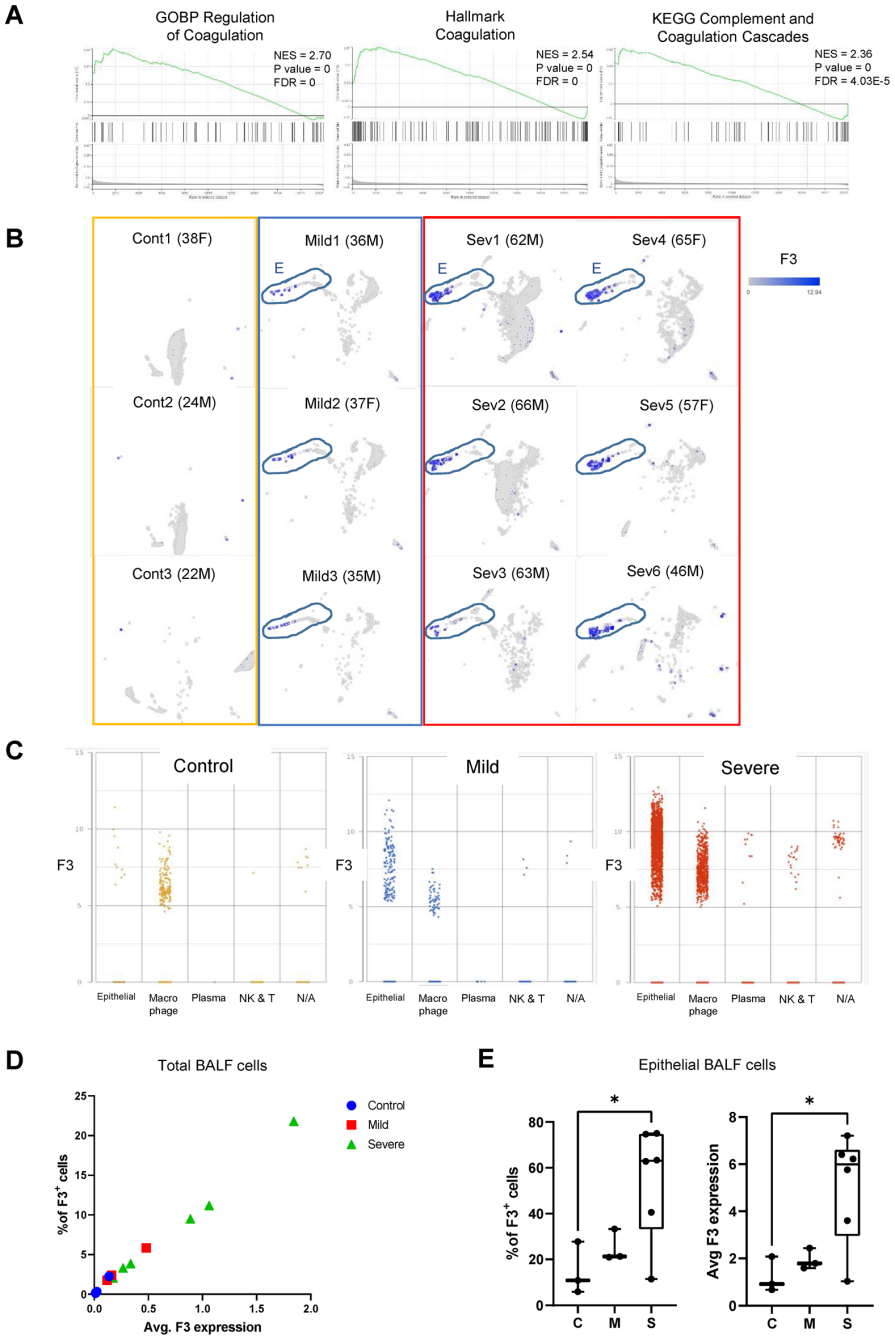
F3 is highly expressed in BALF epithelial cells derived from COVID-19 patients. (**A**) GSEA plots of GOBP coagulation pathways in BALF cells from COVID patients compared to those from healthy control^18^. (**B**) UMAP representation of F3 expression in control, mild, and severe COVID patients. Each gray dot represents a single cell. F3 expression levels are marked with blue color. Epithelial cells are outlined. (**C**) Dot plot of F3 levels across the major cell types within BALF cell populations. (**D**) Plot of the percentage of F3-positive (F3^+^) cells and average F3 expression level (arbitrary unit) in BALF cell populations. Each point represents one patient. (**E**) % of F3^+^ cells and average F3 expression in the BALF epithelial cell populations. Each point represents one patient. * *p* value < 0.05.

Thrombosis and hyper-coagulation are prominent responses to SARS-CoV-2 infection^8,9^. GSEA analysis of BALF cell population derived from COVID-19-infected patients indicates that coagulation pathways are highly enriched in COVID-positive patients (Fig. 2a, Table 1). Tissue factor (F3) is a critical initiator of extrinsic coagulation pathway and is associated with hypercoagulation in various diseases^20^. We tested whether F3 is upregulated upon COVID-19 infection, using the single-cell RNA profiles of BALF samples. High levels of *F3* mRNA expression were found only in COVID-19 patients (Fig. 2b). Furthermore, BALF epithelial cells derived from severe COVID-19 patients showed particularly strong upregulation of F3 (Fig. 2b). To determine the *F3* mRNA expression levels in different cell types within the BALF cell populations, we plotted single-cell expression of F3 against their cell types. We found that F3 is highly upregulated in epithelial cells, in addition to macrophages to a lesser degree (Fig. 2c). This is consistent with previous reports that F3 is primarily expressed in epithelial cells, platelets, fibroblasts, and endothelial cells^21,22^.

To determine if this increase in F3 expression is prominent on a patient level, we calculated both the expression levels of F3 and the percentage of F3-positive (F3^+^) cells per patient. The percentages of F3^+^ cells and F3 levels are significantly higher in severe patients, compared to those in healthy control individuals (Fig. 2d). Additional analysis of epithelial BALF cells showed that severe COVID patients had 40 to 70% of F3^+^ BALF epithelial cells, which is significantly higher than the healthy controls or mild COVID patients (Fig. 2e).

To determine if F3 upregulation is a common event upon SARS-CoV-2 infection, we analyzed additional datasets. First, we determined the F3 expression in SARS-CoV-2 infected Calu3 lung epithelial cells (GSE148729)^23^. In vitro infected samples had a significantly higher F3 expression compared to the control, as early as 1-day after infection (data not shown). We also analyzed the data from SARS-CoV-2 infected golden hamster studies. Three lung samples were taken 1, 2, 4, 6, 8, and 14 days post-infection. In the lung tissue of SARS-CoV-2-infected golden hamsters (GSE161200)^24^, infected lungs showed the highest expression of F3 at the beginning of the infection (data not shown). Taken together, these data support the notion that SARS-CoV-2 infection induces F3 upregulation.

### F3 levels correlate with the senescence and thrombosis signatures in COVID-19

**Figure 3:**
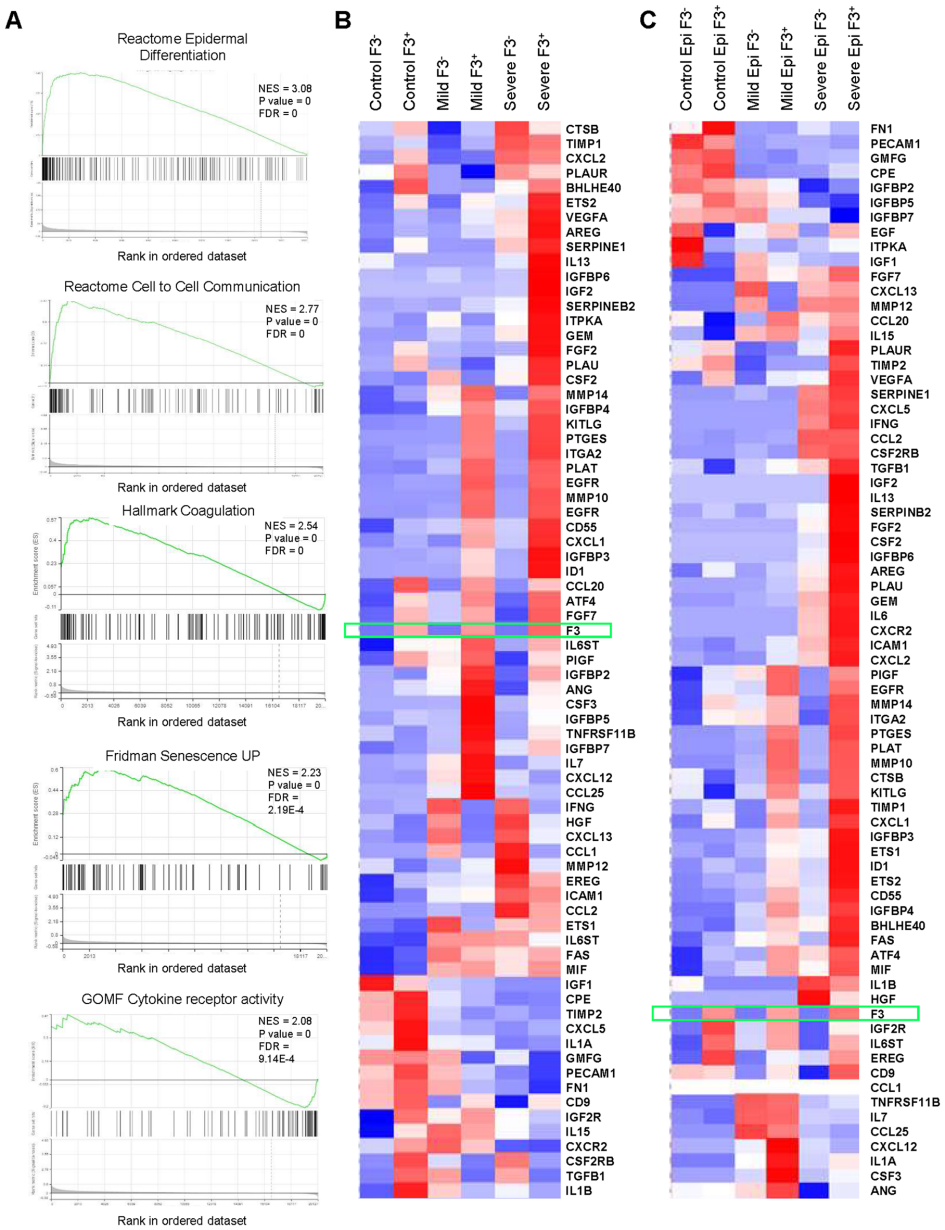
F3^+^ cell populations from COVID patients harbor the upregulated gene signatures for the senescence and coagulation pathways. (**A**) representative GSEA plots for reactome epidermal differentiation, senescence, and coagulation pathways in the F3^+^ populations from COVID patients^18^. (**B**) Heat map plot of the SASP factor expression in the F3^+^ and F3^−^ populations from BALF cells (Severe F3^+^: 2100, Severe F3^−^: 39,223, Mild F3^+^: 201, Mild F3^−^: 8411, Control F3^+^: 180, Control F3^−^: 23,306 cells). (**C**) Heat map plot of the SASP factor expression in the F3^+^ and F3^−^ populations of BALF epithelial cells. (Severe F3^+^: 1454, Severe F3^−^: 1251, Mild F3^+^: 137, Mild F3^−^: 414, Control F3^+^: 10, Control F3^−^: 62 cells).

The above data implicate a strong correlation between F3 level and the senescence phenotype. Comparison of the SASP and F3 expression levels in single-cell plots showed a positive correlation between the two, especially in the BALF epithelial cells derived from severe COVID patients (Figs. 1 and 2). To further investigate the relationship between F3 levels and the senescence signature in COVID infection, we grouped the cells into F3-positive (F3^+^) and F3-negative (F3^-^) subpopulations based on F3 expression and performed GSEA analysis. While the highest-ranked pathway enriched in the F3^+^ BALF epithelial cells from severe COVID patients is the epidermal differentiation signature, other highly enriched pathways include the senescence pathway, inflammatory SASP factors, and coagulation (Fig. 3a). Additional analysis with GSEA and KEGG database showed that F3^+^ BALF cell populations from severe COVID patients have enriched gene signatures for thrombosis and coagulation, as well as tight junction and extra-cellular matrix hallmarks (Fig. 3a, Table 2). Heatmap analysis to determine the levels of representative gene sets including inflammatory cytokines and pro-thrombosis genes further indicates a strong correlation between F3 expression and the upregulation of the senescence and coagulation pathways (Fig. 3b and c).

**Table 2:** KEGG pathway analysis of F3^+^ BALF cells derived from severe COVID-19 patients. Ranked in order of normalized enrichment score (NES).

	**Gene set ID**	**Gene set description**	**Gene set size**	**Enrichment score**	**Normalized enrichment score**	***P* value**	**FDR**
1	path:hsa04530	Tight junction	150	6.22E − 01	2.80E + 00	0.00E + 00	0.00E + 00
2	path:hsa05146	Amoebiasis	98	5.97E − 01	2.67E + 00	0.00E + 00	0.00E + 00
3	path:hsa04512	ECM-receptor interaction	78	6.06E − 01	2.60E + 00	0.00E + 00	0.00E + 00
4	path:hsa05412	Arrhythmogenic right ventricular cardiomyopathy	65	5.90E − 01	2.51E + 00	0.00E + 00	0.00E + 00
5	path:hsa04520	Adherens junction	69	6.48E − 01	2.51E + 00	0.00E + 00	0.00E + 00
6	path:hsa04390	Hippo signaling pathway	139	5.58E − 01	2.48E + 00	0.00E + 00	0.00E + 00
7	path:hsa04510	Focal adhesion	183	5.16E − 01	2.47E + 00	0.00E + 00	0.00E + 00
8	path:hsa05410	Hypertrophic cardiomyopathy (HCM)	76	5.34E − 01	2.43E + 00	0.00E + 00	0.00E + 00
9	path:hsa04360	Axon guidance	164	5.29E − 01	2.41E + 00	0.00E + 00	0.00E + 00
10	path:hsa04670	Leukocyte transendothelial migration	103	5.47E − 01	2.38E + 00	0.00E + 00	0.00E + 00
11	path:hsa04610	Complement and coagulation cascades	69	5.52E − 01	2.38E + 00	0.00E + 00	0.00E + 00
12	path:hsa04915	Estrogen signaling pathway	120	5.49E − 01	2.37E + 00	0.00E + 00	0.00E + 00
13	path:hsa05414	Dilated cardiomyopathy (DCM)	79	5.25E − 01	2.36E + 00	0.00E + 00	0.00E + 00
14	path:hsa04151	PI3K-Akt signaling pathway	307	4.62E − 01	2.34E + 00	0.00E + 00	0.00E + 00
15	path:hsa04918	Thyroid hormone synthesis	62	5.85E − 01	2.34E + 00	0.00E + 00	0.00E + 00
22	path:hsa04350	TGF-beta signaling pathway	81	5.41E − 01	2.26E + 00	0.00E + 00	0.00E + 00
133	path:hsa04218	Cellular senescence	154	4.32E − 01	1.75E + 00	0.00E + 00	1.14E − 02
143	path:hsa04668	TNF signaling pathway	109	4.51E − 01	1.71E + 00	0.00E + 00	1.58E − 02

**Figure 4:**
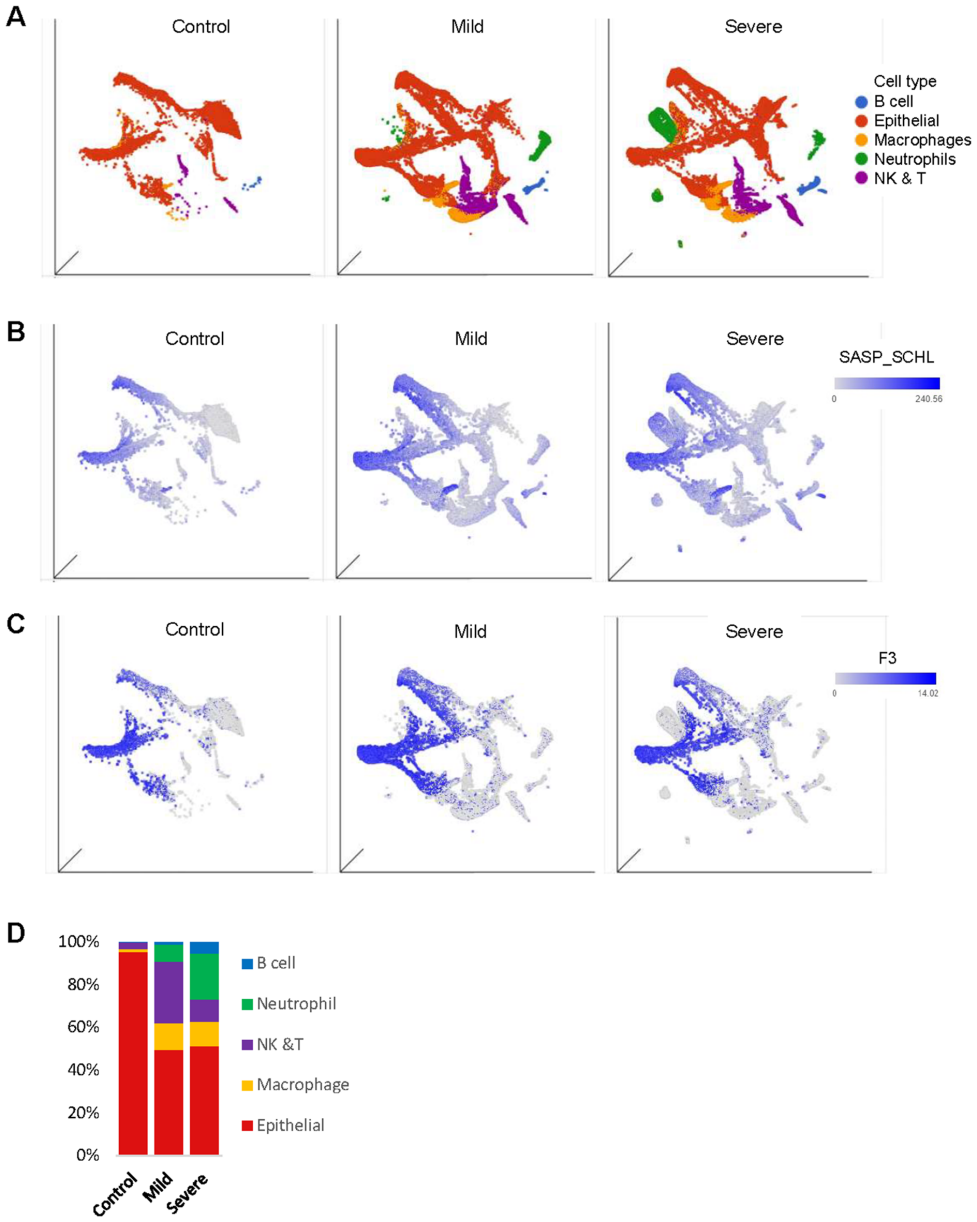
F3 and SASP factor genes are highly expressed in nasopharyngeal (NS) cell population from the COVID-19 patients. (**A**) UMAP projection of the major cell types in NS of COVID-19 patients (control, *n* = 5; mild, *n* = 8; severe, *n* = 11). Cell type composition in NS was quantitated in (**D**). (**B**, **C**) UMAP plot of NS cells derived from healthy control and COVID-19 patients. SASP factor expression level (**B**) and F3 level (**C**) of each cell are marked with blue color.

To confirm the above association further, we conducted a similar analysis using the data from nasopharyngeal swabs (NS) studies^25^. This study consists of NS samples from 24 individuals (5 control, 8 mild, and 11 severe COVID-19 patients). Patients ranged from 21 to 75 years old, of which 11 were admitted to the Intense care unit (ICU), and 2 died from COVID-19^25^. We profiled about 130,000 single cells harvested from the NS swabs and clustered them by cell types (Fig. 4a,b). While most of the control NS samples were primarily epithelial cells, NS cell populations from COVID patients contained more diverse cell types, including neutrophils, macrophages, and natural killer/T cells (Fig. 4b). Similar to the BALF data, we found a strong upregulation of the SASP factor expression and F3 expression in the NS samples from COVID patients (Fig. 4c and d), further indicating a possible link between F3, the senescence, and hyper-coagulation phenotype in COVID infection.

### Potential roles of F3 and SASP in other respiratory viral infections

**Figure 5:**
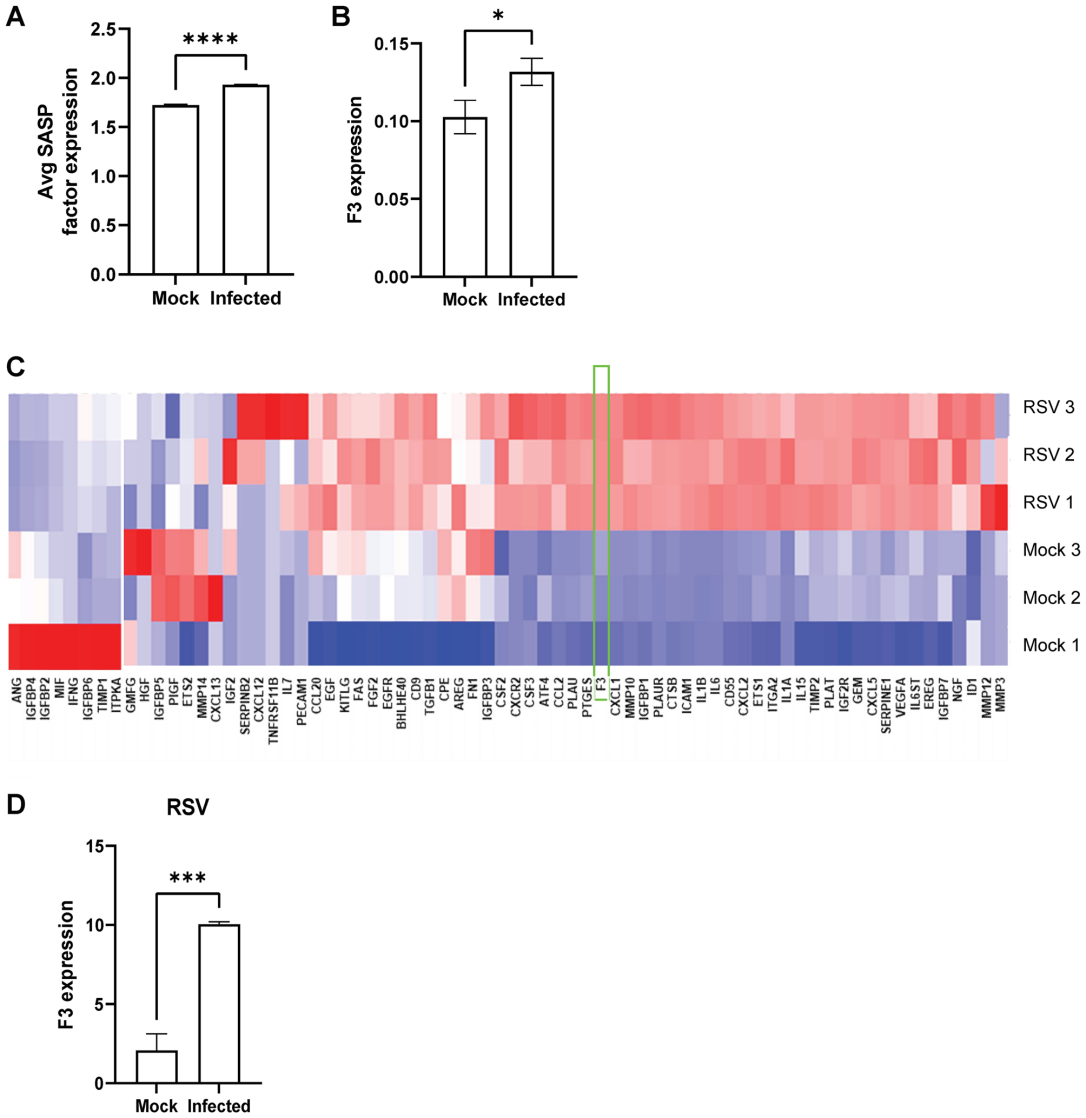
SASP factors and F3 are upregulated upon RSV and IAV infection. (**A**) The levels of representative SASP factor expression in IAV infected A549 cells. (**B**) F3 expression in A549 cells infected with IAV (mock, *n* = 1; IAV *n* = 3). *p value < 0.05. **p value < 0.005. (**C**) Heat map plot of the SASP factor expression in the Mock and RSV-infected A549 cells (*n* = 3 per group). (**D**) F3 expression in RSV-infected A549 cell lines. **p value < 0.005.

Respiratory syncytial virus (RSV) and Influenza A virus (IAV) bear similarities to SARS-CoV-2. For example, RSV and IAV enter the body through airway epithelial cells and they can induce severe inflammation of the upper and lower respiratory systems^26–28^. RSV is one of the most common causes of bronchiolitis and pneumonia in infants, but it can cause cold-like symptoms regardless of age^26^. IAV is a major cause of seasonal flu and it is the only influenza virus known to cause flu pandemics^28^. To investigate whether the senescence phenotype and F3 upregulation are involved in these viral infection models, we analyzed RSV-infected A549 cell studies (GSE147507)^29^ and IAV-infected A549 cell studies (GSE118773)^30,31^. Upon IAV infection, both the SASP factors and F3 expression were significantly upregulated (Fig. 5a and b). We also found a concurrent induction of F3 and the SASP factors upon RSV infection(Fig. 5c and d), suggesting the possibility that the induction of senescence and F3 can be one of common anti-viral responses in humans.

## Discussion

Here, we conducted a meta-analysis of multiple single-cell and bulk-RNA sequencing data and found the upregulation of the senescence phenotype in a broad range of COVID-19 patients. Through a series of bioinformatics-based analyses, we have identified a strong positive correlation between the SASP factor expression, thrombosis, and COVID infection. Through the single-cell RNA data analysis combined with cell type classification, we found that a positive correlation between the senescence feature and COVID infection was particularly prominent in the epithelial cell population. Furthermore, we showed that the senescence pathways were upregulated in other respiratory viruses such as IAV and RSV. Importantly, we identified F3 as a potential link between senescence, inflammation, and thrombosis in infection. Our data collectively indicate that F3 is overexpressed in COVID-19 infection, specifically in infected epithelial cells, and that F3-positive populations have enriched thrombosis and senescence pathways.

Senescence has long been associated with inflammation and cytokine signaling. Increased expression of inflammatory SASP factors such as IL6, MCP1, and CCL2 are known to mediate excessive inflammatory response and cytokine storm of severe COVID-19 patients^11,12^. The wide range of genes involved in senescence programming has resulted in multiple proposed SASP factor lists, which are often quite different from each other^13^. A strong, positive correlation between F3, senescence, and viral infection should be further validated through experimental validation.

F3, the initiator of the extrinsic coagulation pathway, is a protein target that is directly associated with deleterious thrombotic complications in COVID-19. We also found that F3 is overexpressed in COVID-19 infection, specifically in infected epithelial cells. The upregulation of F3 is linked with the increase in D-dimer levels, thrombin, vascular endothelial growth factor, and other clotting factors^20^. The overexpression of F3 and its downstream thrombotic factors may explain the increased thrombosis associated with severe COVID-19 pathologies such as strokes, pulmonary embolism, blood vessel damage, and post-COVID-19 syndrome^20^. Thus, F3 inhibition can be a potential therapeutic target in COVID patients because it can impede hyper-activation of the extrinsic coagulation pathway, hamper the formation of a pro-thrombotic environment, and reduce the vascular damage caused by the hyper-coagulation.

Furthermore, F3 is implicated in the pathologies of other viruses. Similar to SARS-CoV-2 infected cells, we found that RSV and IAV infections demonstrate F3 upregulation and corresponding SASP upregulation. Other studies report that F3 is overexpressed and has a direct correlation with disease progression markers in infections such as Herpes simplex virus, human immunodeficiency virus, and Ebola^32–34^. These findings suggest that F3 upregulation and downstream thrombosis, inflammation, and senescence are common cellular events in other viral infections besides COVID-19. Our findings also suggest that inhibiting F3 may limit the cytokine storm and reduce the formation of a clotting environment to ultimately reduce the severity of COVID-19 and other respiratory viral infections.

Several F3 inhibitors have been developed as anti-thrombotic treatments for various diseases. Ixolaris is a recombinant Tissue factor protein inhibitor (TFPI) that works by binding FXa, which indirectly inhibits FVIIa/TF complex activity^35^. In a murine melanoma model, Ixolaris significantly inhibited coagulation and metastasis^36^. Another F3 inhibitor is recombinant FVIIa with its active site blocked (FVIIai). FVIIai inhibited cyclic flow variations caused by thrombi in rabbit models^37^. Recombinant nematode anticoagulant protein c2 (rNAPc2) works similarly to Ixolaris and blocks the FVIIa active site with a reactive loop to inactivate the FVIIa/TF complex^38^. rNAPc2 has an anti-thrombotic effect, reducing the risk of venous thromboembolism in total knee replacement patients^38^. In another study, rNAPc2-treated Ebola-infected rhesus macaques have prolonged survival, reduced thromboembolisms, and lower plasma concentrations of IL6 and MCP1 than untreated controls^34^. Overall, the targeted, anti-thrombosis effect of F3 inhibitors indicates that F3 inhibitors may have a similar benefit in COVID-19 and other respiratory infections.

Senescence and SASP factors, activated upon conditions like viral infection, exacerbate the biological immune response in affected cells. The resulting clotting, tissue damage, and inflammation are highly associated with mortality in COVID-19. The upregulation of F3 in infected patients support the possibility that F3 mediates an exaggerated SASP release, increases the inflammatory response, and further exacerbates the pro-coagulatory, tissue-damaging environment. Thus, as a link between VIS and the terminal phenotypes observed in severe respiratory viral infections, F3 would be a potential therapeutic target for COVID-19 and related infections. The senolytic capability of an F3 inhibitor has the potential to eliminate the dangerous senescence phenotype and should be pursued as a therapeutic candidate for COVID-19 and other viral infections.

## Methods

### Single-cell data analysis

ScRNA-seq datasets (matrices, barcodes, and genes) were obtained from GEO. BALF data was filtered with the same methods as the source study (only including cells with UMI > 1000, number of genes between 200 and 6000, and mitochondria gene < 0.1%). In the other single-cell datasets, all cells were considered. Then, in all single-cell datasets, genes with ≤ 1 maximum expression were filtered out. The samples were normalized by library size (counts per million) and log transformed. The top 2000 variable genes were identified and used for PCA and UMAP was performed on the top 50 principal components.

### Pseudo-bulk RNA profile

They were generated by pooling the gene expression values of single cells by attribute and calculating the average expression of each gene per cell.

### Bulk RNA-seq data analysis

Datasets were obtained from GEO. Genes with ≤ 1 maximum expression were filtered out and the samples were normalized by library size (counts per million) and log transformed. Z-scores were calculated and represented in heat maps using Partek Flow.

### Cell type classification

Cell type classification was performed by measuring the expression levels of the representative cell type-specific markers; Epithelial (KRT18, TPPP3), Macrophages (CD68), Neutrophil (FCGR3B), and NK/T cells (CD3D, KLRD1) ^17^.

### Gene set enrichment analysis

GSEA was conducted with MSigDB sets with hg38 human assembly. Normalized enrichment scores (NES) with a False discovery rate (FDR) < 0.05 were considered significant.

### Statistical comparisons

For statistical comparisons, ANOVA (2-sided, adjusted via Tukey multiple comparisons test) was used. P values of < 0.05 were considered significant.

## Data Availability

scRNA-seq data from human BALF cells from patients with COVID-19 are available at GEO (GSE145926). Seurat objects of scRNA-seq datasets of nasopharyngeal samples of 19 COVID-19 patients and 5 healthy controls are available at FigShare https://doi.org/10.6084/m9.figshare.12436517. Gene expression data of human AECs at 1–3 days post-infection are available at GEO (GSE148729). Bulk-RNA sequencing datasets of the lungs of golden hamsters infected with COVID-19 are publicly available at GEO (GSE161200). scRNA-seq datasets of A549 cells infected with IAV at mock, 6, 12, and 24 h post-infection are available at GEO (GSE118773). Bulk-RNA datasets of A549 cells infected with RSV are available at GEO (GSE147507).
